# Pre-treatment anxiety in a dental hygiene recall population: a cross-sectional pilot study

**DOI:** 10.1186/s12903-016-0198-8

**Published:** 2016-03-24

**Authors:** Deborah Hofer, Myriam V. Thoma, Patrick R. Schmidlin, Thomas Attin, Ulrike Ehlert, Urs M. Nater

**Affiliations:** Clinic of Preventive Dentistry, Periodontology and Cariology, University of Zurich, Center of Dental Medicine, Plattenstrasse 11, 8032 Zurich, Switzerland; Department of Psychology, University of Zurich, Zurich, Switzerland; Department of Psychology, University of Marburg, Marburg, Germany

**Keywords:** Anxiety, Dental hygiene, Recall

## Abstract

**Background:**

Increased levels of anxiety may affect a patient’s receptiveness to treatment, health care information and behaviour modification.

This study was undertaken to assess pre-treatment anxiety in a dental hygiene recall population maintaining a schedule of regular preventive care appointments.

**Methods:**

The sample population consisted of 46 consecutive adult recall patients waiting for their regularly scheduled dental hygiene appointment. Pre-treatment state (current) anxiety was assessed using the State-Trait-Anxiety Inventory (STAI), State form; dental anxiety with the Hierarchical Anxiety Questionnaire (HAQ); subjective stress using a visual analogue scale (VAS); and mood/alertness/calmness using the Multidimensional Mood Questionnaire (MDMQ).

**Results:**

Two distinct groups, based on state anxiety scores, were formed; one displaying increased levels of pre-treatment anxiety (*n* = 14), the other low anxiety (*n* = 32). The HA group was characterized by significantly higher dental anxiety and subjective stress levels prior to treatment; as well as worse mood, lower alertness, and less calmness in the dental office setting. There was no correlation between anxiety level and years in dental hygiene recall.

**Conclusions:**

A high level of pre-treatment anxiety was present in about one third of the sample population. The prevalence of this anxiety demonstrates the need for both early recognition and patient management strategies (psychological and pain management) to positively influence their treatment experience.

**Electronic supplementary material:**

The online version of this article (doi:10.1186/s12903-016-0198-8) contains supplementary material, which is available to authorized users.

## Background

Dental anxiety may be defined as a feeling of trepidation before visiting a dentist or even contemplating dental procedures [1]. The ramifications of dental anxiety fall on a continuum of patient behaviors ranging from no outward signs of discomfort to avoidance of treatment. Proposed causes for the development of dental anxiety are multifactorial and range from previous negative experiences during dental treatment to psychopathologic personality traits [2–4]. Fear of the unknown, perceived unpredictability of dental treatment and expectation of pain has also been frequently mentioned as causative factors for dental anxiety [5, 6].

In contrast to general dental procedures, routine dental hygiene recall/maintenance involves a relatively narrow range of procedures (recording clinical parameters, oral hygiene evaluation/reinstruction, debridement of soft and hard deposits, fluoride application) that are highly predictable. It may be postulated that the more often a patient attends preventive recall appointments, and the more familiar they become with maintenance procedures, the more likely they are to overcome any pre-treatment anxiety associated with up-coming dental hygiene visits. A study in Finland [7] showed that the subjects in their study who took advantage of dental services on a regular basis were significantly less afraid of going to the dentist that those who only used dental services on an irregular basis. However, other studies on dental anxiety related to dental hygienist treatment have shown that there is still a certain level of anxiety associated with dental hygiene visits [8, 9]. Lazarus postulated in his stress theory [10] that psychological and physiological distress occurs if an individual finds him- or herself confronted with a situation that is uncontrollable and threatening. It is conceivable that patients might feel that a dental hygiene recall/maintenance visit poses a threat sufficient to initiate feelings of stress and anxiety in anticipation of the actual event. However, only very few studies have examined dental anxiety as it is specifically related to dental hygiene maintenance treatment and whether this anxiety is associated with specific clinical, as well as psychological characteristics.

This study was undertaken to measure the anxiety levels of dental hygiene patients who comply with a regular schedule of recall/maintenance therapy appointments. A convenience sample of patients attending a university dental clinic for their regular recall/maintenance dental hygiene appointment were asked to complete a short battery of questions, while waiting for their scheduled appointment to begin. The results were analysed for dental anxiety, expected pain, feelings of stress or relaxation, general mood and alertness pre-treatment, as well as general anxiety felt simply by being in a dental clinic. Previous dental procedures, as depicted on radiographs or from the records, were also noted. We hypothesized that we would observe a substantial amount of pre-treatment anxiety in our sample and this anxiety would be associated with increased levels of expected pain, stress, and worse mood. We expected to find a positive correlation between previous invasive procedures and current levels of anxiety. Further, we hypothesized that there would be a negative correlation between current oral health status, number of recall visits and pre-treatment anxiety.

## Methods

### Study population

This investigation was undertaken at the University of Zurich, Clinic for Preventive Dentistry, Periodontology and Cariology with a convenience sample (*n* = 46) of consecutive recall/maintenance patients waiting for their regularly scheduled dental hygiene appointment. All patients who were at least 18 years of age and displayed a level of German language skills compatible with understanding the survey questions were asked to participate in the study. There were no further exclusion criteria.

### Study design

A random month (March) was chosen for execution of this study. All patients arriving for their regularly scheduled dental hygiene appointments, who fulfilled the inclusion criteria, were asked by a member of the Psychology Department to participate in the study. Those expressing willingness were taken to a separate area of the waiting room, given a detailed explanation of the study, and signed a document of informed consent. All participants were informed that the study was independent of their scheduled treatment and all data would be handled confidentially. The participants were also given a written sheet with the names and telephone numbers of the study supervisors, and informed that they could repeal their consent at any time. The study design had been previously reviewed and approved by the local ethics committee (Kantonale Ethikkommission Zürich, Number: StV 09/11). The patients who declined participation in the study were not documented, to preserve their anonymity and right to refuse without prejudice, as mandated by the ethics commission.

### Patient characteristics

Data routinely gathered during a dental hygiene recall/maintenance appointment (probing pocket depth, bleeding on probing, the presence of hard and soft deposits, carious lesions), plus the patients’ treatment history, was used to establish their periodontal classification [11, 12]. Gingivitis was defined as gingival inflammation with bleeding on probing (BoP) though no loss of attachment and periodontitis was defined as pocket depth/attachment loss of >3 mm and previous active treatment (surgical or non-surgical) for periodontal disease. Further, the patient records and radiographs were reviewed for demographic information, number of years in dental hygiene treatment in the university clinic, as well as previous invasive dental treatment (restorative, endodontic, periodontal and/or surgical). The number of teeth currently present includes 3^rd^ molars, for consistency in reporting, since extraction of any tooth may have been an anxiety-producing event.

### Measurements

#### State-Trait Anxiety Inventory

The State-Trait Anxiety Inventory (STAI) [13] comprise two scales: the Trait and the State Form. Each scale consists of 20 items, with 4 possible responses, that indicate whether anxiety symptoms are present, and to what degree. While the Trait Form measures how threatening one views the world in general, the State Form is a continuous measure for possible changes in current anxiety, considered to be temporary, due to an outside stimulus. Only the State Form was used in this study. The response range for the STAI State Form is 20 (low anxiety) – 80 (high anxiety); clinically relevant anxiety is considered to be scores ≥39. The German language STAI has been validated, with scores between 0.09 and 0.56, [14].

#### Hierarchical Anxiety Questionnaire

The Hierarchical Anxiety Questionnaire (HAQ) [5] is based on the Dental Anxiety Scale [15], and measures anxiety in a dental setting. It contains the most anxiety provoking dental treatment situations (additional file 1), which were distilled from an anxiety hierarchy proposed in a study by Gale [16]. The HAQ is composed of 11 questions, with 5 possible responses and has a response range of 11–55; categorized into the groups low anxiety (up to 30), moderate anxiety (31–38) and high anxiety (over 39 points). The German language HAQ has been validated as it compares to the Dental Anxiety Scale (DAS), and shows good correlation (*r* = 0.88, *p* < 0.01) [17].

#### Visual Analogue Scales

Using visual analogue scales, subjects were asked: a) how stressed they were prior to the treatment session and b) how much pain they expected during the treatment. The visual analogue scales have a response range of 0–100. Visual analogue scales have shown good correlation with STAI (*r* = 0.76, *p* < 0.001) [18] as well as good sensitivity (69.5 %) and good specificity (72.6 %) [19].

#### Multidimensional Mood Questionnaire

The Multidimensional Mood Questionnaire (MDMQ) [20] measures the three dimensions of valence (good vs. bad mood), alertness (awake vs. tired), and calmness (calm vs. nervous) (additional file 2), on a scale ranging from 4–20. Internal consistency (Cronbach’s alpha) of the different dimensions are between α = 0.86 and α = 0.94.

### Statistical analysis

The patients in this study were divided into two groups: a high and a low anxiety group. Allocation to a group was made based on state anxiety levels. Mean differences between the two groups were calculated with Student’s t-tests or chi square tests in case of non-normality of the variables. Correlations between non-normal variables were computed as Spearman’s Rho correlations. For all analyses, the significance level was α = 5 %. The analyses were performed using the Statistical Package for the Social Sciences (SPSS) Version 16.0 (SPSS Inc., Chicago, IL, USA).

## Results

### Patient characteristics

Demographic information of the study participants as well as the time span for which they have been in treatment in the university clinic, the frequency of their dental hygiene appointments during that time span, and the invasive procedures performed on the patients are indicated in Table 1. Of the 167 patients treated in the month selected for study, 46 (24 %) both fulfilled the inclusion criteria and were willing to participate in the study.

**Table 1:** Characteristics of the study population; mean values ± 1 SD and range/percent in brackets, as a whole and divided into high anxiety (HA) and low anxiety (LA) groups

	All participants	LA group	HA group	*p*-value
Age	51.6 ± 17.2	55.5 ± 17.0	42.7 ± 14.7	0.018
	(26–83)	(26–83)	(26–77)	
Number of teeth	25.7 ± 4.2	26.9 ± 4.2	24.5 ± 3.9	0.946
	(13–32)	(14–32)	(13–31)	
Years in treatment at University Clinic	3.7 ± 3.0	3.8 ± 2.9	3.6 ± 3.2	0.895
	(1–11)	(1–9)	(1–11)	
Dental Hygiene appointments, total	7.4 ± 6.9	7.7 ± 6.7	6.7 ± 7.6	0.716
	(1–27)	(1–26)	(1–27)	
Dental Hygiene Appointments/year	3.5 ± 3.2	3.6 ± 3.8	3.3 ± 2.3	0.789
	(1–15)	(1–15)	(1–10)	
Periodontal classification				0.005
Gingivitis	22 (47.8 %)	11 (34.4 %)	11 (78.6 %)	
Periodontitis	24 (52.2 %)	21 (65.6 %)	3 (21.4 %)	
Periodontal Surgery				0.913
Yes	3 (6.5 %)	2 (6.3 %)	1 (7.1 %)	
No	43 (93.5 %)	30 (93.8 %)	13 (92.9 %)	
Implants				0.237
Yes	12 (26.1 %)	10 (31.3 %)	2 (14.3 %)	
No	34 (73.9 %)	22 (68.8 %)	12 (85.7 %)	
Restorations				0.073
None	3 (6.5 %)	3 (9.4 %)	0	
Single per Quadrant	4 (8.7 %)	0	4 (28.6 %)	
Multiple per Quadrant	9 (19.6 %)	4 (12.5 %)	5 (35.7 %)	
Multiple per Quadrant + crowns/bridges	30 (65.2 %)	25 (78.1 %)	5 (35.7 %)	
Extractions				0.324
Yes	39 (84.8 %)	26 (81.3 %)	13 (92.9 %)	
No	7 (15.2 %)	6 (18.8 %)	1 (7.1 %)	
Root canal treatment				0.514
Yes	1 (2.2 %)	1 (3.1 %)	0	
No	45 (97.8 %)	31 (96.9 %)	14 (100 %)	

### State anxiety and patient characteristics

On the average, state anxiety levels for the patients questioned in the waiting room prior to dental hygiene treatment were relatively high (mean = 34.7, SD = 9.7; Table 2). This was close to the recommended cut-off value of 39 for the diagnosis of clinically relevant anxiety. In order to compare individuals with high and low levels of anxiety, two groups were formed: a high anxiety (HA) group (STAI score ≥ 39; *n* = 14, mean = 46.6, SD = 6.9) and a low anxiety (LA) group (STAI score ≤ 38; *n* = 32, mean = 29.5, SD = 5.0). The two groups did not differ regarding sex distribution, total number of dental hygiene appointments visited or average number of dental hygiene appointments per year (comparisons not significant). However, the LA group was older than the HA group (*p* = 0.018). Further, the two groups did not differ in regards to prior dental treatment (extractions, periodontal surgeries, implants, root canal treatments and/or restorations). However, the HA group had more gingivitis and the LA group had more periodontitis (*p* = .005).

**Table 2:** Mean values of the respective anxiety scales ± 1SD and range in brackets, for the study population as a whole and divided into high anxiety (HA) and low anxiety (LA) groups

	All participants	LA group	HA group	*p*-value
STAI state (current) anxiety	34.7 ± 9.7	29.5 ± 5.0	46.6 ± 6.9	<0.001
	(20–64)	(20–38)	(39–64)	
HAQ dental anxiety	23.5 ± 8.6	20.2 ± 6.3	30.1 ± 8.9	<0.001
	(11–55)	(11–35)	(21–55)	
VAS anticipated pain	26.7 ± 24.2	22.9 ± 23.7	34.7 ± 24.1	0.134
	(0–87)	(0–87)	(0–80)	
VAS current stress level	26.1 ± 29.0	18.2 ± 26.2	43.5 ± 27.9	0.005
	(0–98)	(0–98)	(0–77)	
MDMQ current mood	17.0 ± 3.1	18.1 ± 2.2	14.2 ± 3.1	<0.001
	(9–20)	(12–20)	(9–18)	
MDMQ wakefulness	15.1 ± 4.0	16.5 ± 3.5	11.8 ± 3.0	<0.001
	(5–20)	(5–20)	(8–17)	
MDMQ calmness	16.0 ± 3.3	17.3 ± 2.2	12.9 ± 3.5	<0.001
	(7–20)	(13–20)	(7–18)	

### Dental anxiety

The two groups differed significantly regarding general dental anxiety (LA: mean = 20.2, SD = 6.3; HA: mean = 30.1, SD = 8.9; *t*_40_ = −4.15, *p* < 0.001).

### Anticipated pain and stress

The two groups did not significantly differ regarding anticipated pain during treatment (LA: mean = 22.9, SD = 23.7; HA: mean = 34.7, SD = 24.1; *t*_41_ = −1.53, *p* = 0.134). There were, however, significant differences in their current stress levels (LA: mean = 18.2, SD = 26.2; HA: mean = 43.5, SD = 27.9; *t*_43_ = −2.94, *p* = 0.005).

### Current mood

LA and HA also differed regarding their current mood prior to the treatment. LA individuals were in a significantly better mood (LA: mean = 18.1, SD = 2.2; HA: mean = 14.2, SD = 3.1; *t*_38_ = 4.67, *p* < 0.001), were more awake (LA: mean = 16.5, SD = 3.4; HA: mean = 11.8, SD = 2.9; *t*_38_ = 4.67, *p <* 0.001), and calmer compared to the HA group (LA: mean = 17.3, SD = 2.2; HA: mean = 12.9, SD = 3.5; *t*_38_ = 4.71, *p* < 0.001).

### Anxiety and recall visits

Correlations were computed between anxiety levels and either number of visits or years participants had been on recall. No significant correlations were found (number of visits: *r* = −0.086 for HAQ and *r* = −0.092 for STAI, respectively; years participants had been on recall: *r* = −0.089 for HAF and *r* = 0.122 for STAI, respectively).

## Discussion

The hypothesis that pre-treatment anxiety would be substantial in our sample population and that this anxiety would be associated with increased levels of stress and worse mood was confirmed by the results. Expectation of pain, however, was not proven, though a tendency was discernable. The hypothesis that there would be a negative correlation between number of recall visits and pre-treatment anxiety was rejected.

The goal of this study was to assess to what degree pre-treatment anxiety may be found in a dental hygiene recall population. The patients in our sample population had also been treated previously in one or more of the university dental clinic specialty programs: restorative, periodontics, endodontic, prosthetic. The dental hygiene treatment that the subjects were waiting for consisted of recording plaque/bleeding indices, oral hygiene instruction, deplaqueing and light calculus removal.

A convenience sample of patients waiting for their dental hygiene recall appointment during a random month served as the basis for this pilot study. The results show that about one third of the participating patients reported substantial levels (above the clinically relevant cut-off level 39) of pre-treatment state anxiety. Therefore, the population was divided into sub-groups HA and LA, whereby further statistically significant group differences between the group results for dental anxiety, current stress levels, current mood, wakefulness, calmness were found. State anxiety also served as the dependent variables against which the variables of age, years in treatment, appointments per year, periodontal classification and the more invasive treatments listed in Table 1 could be tested for correlation.

Further examination of the characteristics of the HA subjects shows that the group is significantly younger than the group with less anxiety (average age: 42 vs 55 years). This supports the findings of two recent Finnish studies, where the results showed that the percentage of subjects who were very or somewhat afraid of visiting a dentist was higher among younger age groups (41–52 % of subjects age 30–49 vs 16–28 % of subjects age 50–65+) regardless of how regularly or irregularly they sought treatment [7, 21]. Also, in an early study by Corah et al. [22], younger subjects exhibited higher dental anxiety scores than subjects who were somewhat older (difference: 8 years). In all three studies, where the results appear to show a trend for anxiety to recede with age, it did not disappear completely.

The HA group also reported higher pre-treatment stress levels, and tended to anticipate more pain during treatment than the LA group. These findings are very similar to the results reported in earlier studies [4, 23] and are attributed by numerous authors to painful procedures (probing, scaling, administration of anaesthesia), contextual stimuli (sound of the ultrasonic and polishing hand-pieces, vibration sensation on the teeth, dental office smell) and past dental/dental hygiene experiences [24–26].

In our study, however, procedures that could be classified as painful (or at least invasive) such as implant placement, periodontal surgery, multiple restorations, extractions and root canal treatment were not correlated with increased anxiety. Interestingly, aside from age, gingivitis was the only other correlate to increased anxiety levels. As plaque-induced gingivitis is often the result of life style choices (inadequate removal of soft deposits on the teeth on a daily basis), it may be that for these patients dental anxiety causes ambivalence about maintaining optimal oral hygiene, even if they maintain a schedule of regular dental hygiene recall appointments [27].

In addition to behavioural issues associated with pre-treatment stress levels (not being receptive to information, lack of cooperation in treatment, ambivalence about home care modification, delaying/avoiding treatment) [28], current understanding of periodontal disease implicates stress as a risk factor for both inflammation and delayed healing. Often cited sources of stress include job, financial, relationship and health [29]. To date, pre-treatment anxiety has not been mentioned as a possible correlate of stress. However, further investigation along this line may be warranted, also in light our findings that the HA group displayed significantly more gingivitis than did the LA group.

Despite not being able to pinpoint why so many patients suffer a relevant degree of anxiety prior to dental hygiene recall treatment, it remains incumbent upon the treating hygienist to help these patients overcome negative feelings associated with preventive/maintenance therapy. Dental phobia and anxiety appear to have remained constant over the past 50 years [23], despite advances in technology and treatment delivery. It does not appear that the numerous coping strategies set forth in the literature have either been provided to and/or have had much of a positive impact on a subgroup of patients suffering from substantial pre-treatment anxiety.

Herein lies the rational for this pilot study; members of the university psychology department were interested in testing an intervention that they postulated would reduce anxiety in patients waiting for minor routine medical treatment. It is not in the scope of this paper to debate whether dental hygiene maintenance/recall treatment should be classified as minor routine treatment, but the pre-test aspect of their planned study was of great interest: was there pre-treatment anxiety in our patient population when waiting for treatment by the dental hygienist? Based on the results of this pilot study, it was determined that such anxiety was present in a relevant portion of our patient population and that our patients may benefit from their planned intervention [30].

Although not part of the study design, our experience with allowing the psychology department members to screen our patients prior to treatment resulted in treatment delays of up to 20 min. Further, when the intervention technique was tested, an additional 10 min were required. In this case, the patients who agreed to participate in the study were willing to take the extra time into account. The treating hygienists, however, remarked that the patient screenings for dental anxiety placed an additional time pressures that made adherence to their treatment schedule difficult. Average dental hygiene appointment times are 1 h per visit. With patients also quoting time and cost as reasons for avoidance of treatment [26], anxiety reducing intervention would need to be short, preferably self-administered and/or run concurrent to the scheduled dental hygiene recall treatment.

In addition to psychological interventions, pharmacological pain management may also offer some benefit when treating anxious patients [31]. Previous studies have shown that patients with HA have a chronic tendency to expect, and remember, more pain than that which they ultimately experience [32, 33]. Further, should they experience more pain than originally anticipated, they will then not only anticipate even higher levels of pain for the next treatment but their increased fear level will be long-lasting [34, 35]. The results of our current study showed a tendency toward higher levels of anticipated pain by the HA subjects. When a cycle of anticipated pain and pre-treatment anxiety exists, helping patients maintain consistently lower-than-anticipated pains levels may be a necessary step in reorganizing their expectations and facilitating a decrease in their anxiety levels. In view of this, pain management should remain a top priority when treating HA patients.

Given these findings, one limitation of the present study was that we did not assess whether pain levels were chronically high in our subjects. Increased pain ratings over time might have an impact on current pain ratings, so future studies should account for this important factor. Another limitation of our study was that the sample was relatively small. In this light, our results have to be considered as pilot findings and need to be replicated in a larger sample. An increased sample size would also help compensate for any bias that may be present by not taking into account those patients unwilling to participate. In addition, the patients participating in this study were originally referred to our clinic for specialty (complicated) treatment or came on their own due to unsatisfactory experiences in private dental offices. We have no record of events that happened in the past, and can make no correlation to their current feelings concerning the dental hygiene treatment provided in our clinic.

## Conclusions

In summary, it can be said that a significant degree of pre-treatment anxiety was present in about one third of the sample population. Although these patients continue to seek dental hygiene treatment, despite negative feelings, the prevalence of this anxiety is a factor for consideration in treatment delivery. Early recognition of HA patients, as well as pain management, are integral steps in ensuring a positive treatment experience. Further research is needed, however, to validate this pilot study’s findings and to identify and develop short, practical and effective psychological interventions that would alleviate anxiety and stress in this patient population, while not placing additional time demands on the dental hygienist, thereby increasing the likelihood of screening and intervention(s) actually being implemented.

## Additional files

Additional file 1: Hierarchical Anxiety Questionnaire (HAQ)*, with the distribution of answers, translated into English. (DOC 112 kb) Additional file 2: MDMQ – Short Form A, translated into English. (DOC 55 kb)

## Abbreviations

HA: high anxiety
HAQ: Hierarchical Anxiety Questionnaire
LA: low anxiety
MDMQ: Multidimensional Mood Questionnaire
STAI: State-Trait-Anxiety Inventory
VAS: visual analogue scale
