# Supplementary material for: Pre-treatment anxiety in a dental hygiene recall population: a cross-sectional pilot study
Source: BMC Oral Health. 2016 Mar 24;16:43. doi: 10.1186/s12903-016-0198-8 (PMC4806470; doi:10.1186/s12903-016-0198-8)
Supplement: Additional file 1: — Hierarchical Anxiety Questionnaire (HAQ)*, with the distribution of answers, translated into English. (DOC 112 kb) [file 12903_2016_198_MOESM1_ESM.doc]

| **Hierarchical Anxiety Questionnaire (HAQ)*,**  with the distribution of answers, translated into English | | | | | |
| --- | --- | --- | --- | --- | --- |
|  | Relaxed | Nervous | Tense | Anxious | Nauseous from anxiety |
| How do you feel when you imagine you have to go to the dentist tomorrow? |  |  |  |  |  |
| You are sitting in the waiting room and are waiting to be called. How do you feel? |  |  |  |  |  |
| Imagine you are entering the room where treatment will be provided and you can smell the typical odours. |  |  |  |  |  |
| You are lying in the dentist’s chair and the dentist enters the room. |  |  |  |  |  |
| You and your dentist are looking at the X-rays and discussing what work needs to be done. |  |  |  |  |  |
| How do you feel when your dentist tells you that he or she will now clean the tartar off your teeth? |  |  |  |  |  |
| The dentist tells you that you have a cavity and that he or she will now treat it. |  |  |  |  |  |
| The dentist changes the position of your chair and prepares an injection. |  |  |  |  |  |
| Imagine you hear the typical sound of a dentist’s drill. How do you feel? |  |  |  |  |  |
| The dentist tells you that the cavity is too deep and the tooth must be removed. |  |  |  |  |  |
| One of your wisdom teeth is to be removed; the injection has already been given. The dentist picks up the scalpel. |  |  |  |  |  |

Please take your time and imagine you are in the situations described above, and then place an “X” in the box of the questionnaire corresponding to how you feel.

Point values for each category: 1 2 3 4 5

Possible range of scores: 11 – 55 points

Total score categories: low anxiety ≤30 points

Moderate anxiety 31-38 points

High anxiety ≥39 points

*Translation of the original German version [5]
