# Supplementary material for: Pre-treatment anxiety in a dental hygiene recall population: a cross-sectional pilot study
Source: BMC Oral Health. 2016 Mar 24;16:43. doi: 10.1186/s12903-016-0198-8 (PMC4806470; doi:10.1186/s12903-016-0198-8)
Supplement: Additional file 2: — MDMQ – Short Form A, translated into English. (DOC 55 kb) [file 12903_2016_198_MOESM2_ESM.doc]

**MDMQ – Short Form A,**

translated into English

| Date and Time: |  |
| --- | --- |

**Instruction**

In the following you find a list of expressions that characterize different moods. Please take a look at the list, word by word, and mark for each word the answer that represents best the actual intensity of your mood status.

Right now I feel...

|  | definitely not  1 | 2 | 3 | 4 | extremely  5 |
| --- | --- | --- | --- | --- | --- |
| 1. content | 🞆 | 🞆 | 🞆 | 🞆 | 🞆 |
| 2. rested | 🞆 | 🞆 | 🞆 | 🞆 | 🞆 |
| 3. restless | 🞆 | 🞆 | 🞆 | 🞆 | 🞆 |
| 4. bad | 🞆 | 🞆 | 🞆 | 🞆 | 🞆 |
| 5. worn-out | 🞆 | 🞆 | 🞆 | 🞆 | 🞆 |
| 6. composed | 🞆 | 🞆 | 🞆 | 🞆 | 🞆 |
| 7. tired | 🞆 | 🞆 | 🞆 | 🞆 | 🞆 |
| 8. great | 🞆 | 🞆 | 🞆 | 🞆 | 🞆 |
| 9. uneasy | 🞆 | 🞆 | 🞆 | 🞆 | 🞆 |
| 10. energetic | 🞆 | 🞆 | 🞆 | 🞆 | 🞆 |
| 11. uncomfortable | 🞆 | 🞆 | 🞆 | 🞆 | 🞆 |
| 12. relaxed | 🞆 | 🞆 | 🞆 | 🞆 | 🞆 |

Please pay attention to the following facts:

* Within the list there are some attributes that possibly describe the same or similar moods.

Please do not get irritated due to this fact, and judge each attribute irrespective of your

answer to another attribute.

* Please judge only how you feel at this moment, and not how you normally or sometimes feel.

* If you have some difficulties in finding an answer, please mark that answer that fits best.

Please judge each word and do not leave out any words.

*Translation of the German version [12]
